# How Consumers Transition Between Illicit and Legal Cannabis Markets in Early Legalization Periods? Evidence from a National Panel with a Recent Policy Shock

**DOI:** 10.21203/rs.3.rs-9381647/v1

**Published:** 2026-04-14

**Authors:** Lei Xu, Shiqi Zhang, Kai Su, Ce Shang

**Affiliations:** 1 Center for Tobacco Research, The Ohio State University Comprehensive Cancer Center, The Ohio State University, 3650 Olentangy Rd., Columbus, OH 43214, USA; 2 Department of Agricultural, Environmental, and Development Economics, The Ohio State University, 2120 Fyffe Rd., Columbus, OH 43210, USA; 3 Department of Human Sciences, The Ohio State University, 1787 Neil Ave., Columbus, OH 43210, USA; 4 Division of Medical Oncology, Department of Internal Medicine, College of Medicine, The Ohio State University Wexner Medical Center, 460 W 10^th^ Ave., Columbus, OH 43210, USA

**Keywords:** Recreational cannabis legalization, Illicit market, Legal sourcing, Difference-in-differences, Ohio legalization

## Abstract

**Introduction:**

States across the US continue to legalize recreational cannabis to shift consumers from illicit to regulated markets. Yet evidence on whether legalization increases the share of cannabis obtained legally remains limited. This study examines how legalization influences cannabis sourcing behavior using nationally representative panel data and recent policy variation.

**Methods:**

We use three waves of panel survey data collected in June 2024 (pre-legalization), January 2025, and August 2025. The analytic sample is a balanced panel of 578 adults (1,734 observations). We estimate difference-in-differences models of the proportion of cannabis obtained from legal sources, exploiting Ohio’s August 2024 legalization within a multi-state context. Models control for baseline policy status, demographics, and wave fixed effects.

**Results:**

Legal sourcing varies substantially across policy environments. Individuals residing in states with fully legal cannabis markets report significantly higher reliance on legal sources than those in fully illegal states (31.6 percentage points, p<0.001). Exploiting the timing of Ohio’s legalization, we find a 5.3 percentage-point increase in the share of cannabis obtained from legal sources (p<0.01), although estimates are sensitive to sample restrictions. Legal sourcing is higher among more educated individuals and lower among Black non-Hispanic respondents.

**Discussion:**

These findings suggest legalization can shift cannabis purchasing from illicit to legal markets in the short run, but the transition is incomplete and heterogeneous. By directly measuring legal sourcing, this study provides new evidence on substitution between illicit and regulated markets. The results highlight the role of broader policy and market conditions in shaping consumer responses.

## 1. INTRODUCTION

Recreational cannabis remains illegal under U.S. federal law; however, states have increasingly exercised their authority to legalize and regulate cannabis within their own jurisdictions^1,2^. Since the early 2010s, state-level legalization has expanded rapidly, with most adoptions occurring in the past decade and varying widely in timing and regulatory design across states^2^. As of 2025, recreational cannabis is legal in 24 states and the District of Columbia, 21 of which adopted legalization after 2014, Colorado and Washington enacted recreational legalization in 2012, followed by Alaska, Oregon, and the District of Columbia in 2014, reflecting a sharp acceleration of reform over the past decade^3,4^. Legal cannabis markets have expanded in parallel, with U.S. legal cannabis sales exceeding $35 billion in 2024, up from less than $5 billion in 2014, reflecting the rapid growth of regulated markets alongside the continued presence of illicit supply^5,6^.

A central motivation behind these reforms is the expectation that legalization will transition consumers away from illicit markets toward regulated legal systems. Legal markets enable governments to impose product standards, enforce age restrictions, and monitor distribution channels, while also generating tax revenue through excise and sales taxes^7–9^. In contrast, illicit cannabis markets operate outside regulatory oversight, where products may be subject to contamination and uncertain quality, thereby limiting consumer protection and undermining public health objectives^10–12^. These regulatory and fiscal objectives depend critically on whether consumers actually transition their purchases from illegal sources to licensed retailers^13^. If a substantial share of demand remains in the illicit market, the anticipated public health and regulatory benefits of legalization may be undermined^9^.

Despite the policy importance of this transition, empirical evidence on whether and to what extent legalization induces substitution from illicit to legal cannabis remains limited, particularly in the short run following policy adoption. Much of the existing literature focuses on changes in cannabis prevalence, frequency, and initiation after legalization, often using difference-in-differences or repeated cross-sectional designs, and generally reports modest increases in adult use alongside limited or heterogeneous effects on youth consumption^14–19^. However, far fewer studies directly examine consumers’ sourcing decisions or quantify individual-level switching from illegal to legal cannabis markets, and the limited evidence that does exist is largely based outside the United States^12^. For example, prior work suggests that legalization alone may be insufficient to fully displace illicit markets when legal products are perceived as more expensive or less aligned with consumer preferences^12^. Other studies have explored how legalization shifts in consumer attitudes and purchasing preferences toward legal versus illegal products^20–22^, cannabis legal versus illegal markets^23^, and spillover effects on co-use with other substances, such as cigarettes^24^. As a result, direct evidence on short-run substitution between illicit and legal cannabis sources in the U.S. context remains scarce.

Using detailed survey data that directly capture both legal and illegal cannabis purchases, this study provides a unique opportunity to estimate shifts in purchasing channels following legalization and to quantify individual-level substitution from illicit to legal markets. We build on prior evidence that has examined substitution between legal and illegal cannabis products, such as substitution between legal flower and illegal flower in response to tax structures, but has largely relied on experimental designs or hypothetical choice settings rather than observed post-legalization purchasing behavior^25^. We leverage three waves of panel data and exploit recent policy variation, including Ohio’s August 2024 legalization, as a natural experiment within a broader multi-state setting^26–28^. This approach allows us to estimate shifts in purchasing channels following legalization and to quantify individual-level substitution from illicit to legal markets. By directly measuring the proportion of cannabis obtained from legal sources, our study contributes new evidence on short-run substitution dynamics that have been difficult to observe in prior research.

## 2. METHODS

### 2.1 Data

This study uses data from three waves of a nationally representative survey of U.S. adults aged 21 and older who use cannabis, collected through the Ipsos Public Affairs KnowledgePanel. The first wave was carried in June 2024, while the second and third wave were in January 2025 and August 2025, respectively. We define the pre-treatment period as June 2024 and the post-treatment period as January and August 2025. This timing allows us to exploit recent policy variation, including Ohio’s August 2024 legalization, within a broader multi-state context. Across all three waves, Ipsos invited one adult per sampled household to participate in the survey. Eligibility criteria were consistent across waves and included: (1) being 21 years of age or older, (2) residing in the United States, and (3) reporting recreational cannabis use in the past 30 days. The analytical sample included 1,734 participants, including 578 participants per wave.

The survey collected detailed information on respondents’ real-world cannabis consumption and purchasing behaviors, including product types used and purchased (e.g., flower, pre-rolls, edibles, cartridges, and concentrates), quantities, and expenditures. Importantly, respondents reported the percentage of cannabis obtained from legal sources, which allows us to directly measure reliance on legal versus illegal or informal markets rather than inferring sourcing from indirect proxies. The survey also collected rich sociodemographic information, including age, gender, race and ethnicity, household income, employment status, and state of residence.

### 2.2 Measures and Variable Definitions

#### 2.2.1 Outcome Variable

The primary outcome variable is the proportion of cannabis obtained from legal sources, measured at the individual–wave level. Respondents were asked to report the percentage of their total cannabis purchases in the past 6 months that were obtained from legal retail sources, with the remainder implicitly sourced from illegal or informal channels. This continuous measure ranges from 0 to 100 and directly captures reliance on the legal market.

#### 2.2.2 Treatment and Policy Variables

The key policy variable of interest is an indicator for Ohio recreational cannabis legalization. We define *Treat*_*i*_ as an indicator equal to 1 if individual *i* resides in Ohio and 0 otherwise. The post-legalization indicator, *Post*_*t*_, equals 1 for observations collected after the implementation of recreational cannabis retail sales in Ohio (January 2025 and August 2025 survey waves) and 0 for the pre-legalization period (June 2024). The interaction term *Post*_*t*_ × *Treat*_*i*_ captures the average change in legal sourcing associated with legalization on the proportion of cannabis sourced legally.

To account for baseline differences in policy environments across states, we include an indicator for pre-treatment state legalization status measured at 2024 June. States are classified into three mutually exclusive categories: (1) fully illegal, (2) mixed policy (medical cannabis legal but recreational cannabis illegal), and (3) fully legal (both medical and recreational cannabis legal). These indicators control for pre-existing access to legal cannabis markets that may influence baseline sourcing behavior.

#### 2.2.3 Demographic and Socioeconomic Covariates

All regression models control for a rich set of individual-level demographic and socioeconomic characteristics measured consistently across waves. These include age (in years), gender (male or female), race and ethnicity (White non-Hispanic; Black non-Hispanic; Hispanic; other non-Hispanic; and two or more races non-Hispanic), marital status (married; not married), educational attainment (no high school diploma/GED; high school graduate; some college or associate degree; bachelor’s degree; and master’s degree or higher), and employment status (working full-time; working part-time; or not working). These covariates are included to account for systematic differences in cannabis sourcing behavior associated with socioeconomic status and demographic characteristics.

### 2.3 Analytical Methods

This paper examines how cannabis legalization affects the proportion of cannabis obtained from legal sources, leveraging variation in policy environments across states. We exploit the timing of Ohio’s August 2024 legalization as a policy shock within a broader multi-state setting. Inspired by a difference-in-differences framework, we estimate the following model:

$$y_{it}=\beta_{1}{\text{Post}}_{t}+\beta_{2}{\text{Treat}}_{i}+\beta_{3}{\text{Post}}_{t}\times {\text{Treat}}_{i}+\beta_{4}\text{Legal}2024_{i}+X_{it}\gamma +\alpha_{t}+\epsilon_{it}$$

where *y*_*it*_ is the share of cannabis consumption from legal sources of individual *i* reported in wave *t*. *Post*_*t*_ is the index of it, which equals 1 if the observation is in wave 2 and 3, and 0 otherwise. *Treat*_*i*_ is the index of treatment group, which is one if the individual *i* is from Ohio, and zero otherwise. The coefficient of interaction term *β*_*3*_ captures the change in legal sourcing following legalization in Ohio relative to contemporaneous changes in other states, conditional on baseline policy differences. The primary analysis uses the full national sample to maximize statistical power and external validity, while subsample analyses are used as robustness checks.

The state legalization status at the beginning of 2024, *Legal2024*_*i*_, is included to control for the starting policy condition. *X*_*it*_ represents the demographic characteristics of individuals, including age, gender, race and ethnicity, education level, marital status, and employment. *α*_*t*_ is the wave fixed effect. Because the data is a short panel with only three waves, including individual fixed effects will lead to potential collinearity and very little within variation, we include individual characteristics instead.

To examine the heterogeneity, we apply the regression on three different samples. The benchmark results are based on the full sample, in which *β*_*3*_ shows the policy effect relative to all the other states. Additionally, we restrict the sample to only individuals from neighborhood states (Pennsylvania, Indiana, West Virginia, Kentucky, Michigan, and Illinois, together with Ohio) to account for potential regional factors. We do the third test based on the individuals from states with mixed cannabis policy at wave 1, i.e., states where recreational use was not legal but medical use was legal at wave 1.

## 3. RESULTS

Our final analytic sample is a strictly balanced panel consisting of 1,734 observations from 578 individuals followed across three survey waves). Table 1 presents summary statistics for the our study variables. On average, participants reported obtaining 77.6% of their cannabis from legal sources. The sample reflects substantial variation in policy environments across states, with 67.9% of observations from fully legal states, 25.9% from medical-only states, and 6.2% from fully illegal states. A small share of observations (4.2%) correspond to individuals residing in Ohio, which experienced legalization during the study period. The analysis leverages variation across all states, with Ohio representing a subset of observations within the national sample.

Additional demographic characteristics are presented in Table 1. Participants ranged in age from 21 to 83 years, with a mean age of 50.2. Approximately 61% of the sample was male. The majority of respondents identified as White non-Hispanic (76.8%), while Hispanic and Black non-Hispanic individuals each accounted for about 10% of the sample. Marital status was evenly distributed, with roughly half of participants married (49.3%) and half not married (50.7%). Educational attainment was relatively high, with 63.2% of respondents reporting at least some college education or a bachelor’s degree. In terms of employment, 53.7% of participants were employed full-time, 13.8% worked part-time, and 32.5% were not working.

Table 2 presents the benchmark difference-in-differences regression results. Legal sourcing varies substantially across policy environments, with individuals residing in states where recreational cannabis was already legal prior to treatment reporting significantly higher reliance on legal sources than those in fully illegal states, with increases of 31.6–33.6 percentage points (with controls: β = 31.65, p < 0.001; without controls: β = 33.63, p < 0.001). Individuals in states with mixed cannabis policies also exhibit higher legal sourcing (β = 11.78, p < 0.05).

Exploiting the timing of Ohio’s legalization within this broader multi-state context, we find an approximately 5.3 percentage-point increase in the proportion of cannabis obtained from legal sources (β = 5.31, p < 0.001). This estimate is consistent across model specifications. Column (1) includes only pre-treatment legalization status and wave fixed effects, while Column (2) further incorporates individual-level demographic controls. In both specifications, the interaction between the post-legalization period and Ohio residency remains positive and statistically significant, and standard errors are clustered at the state level. Given the relatively small number of Ohio respondents, however, this estimate should be interpreted with caution.

Demographic patterns further indicate unequal reliance on legal cannabis markets (see Appendix Table A.1). Black non-Hispanic respondents report significantly lower legal sourcing than White non-Hispanic respondents (*β* = −16.94, p < 0.01), whereas respondents identifying as other non-Hispanic report higher legal sourcing (*β* = 8.82, p < 0.001). Educational attainment is positively associated with legal sourcing. Compared with individuals without a high school diploma, legal sourcing is 15.68 percentage points higher among high school graduates (*β = 15.68*, p < 0.05), 19.3 percentage points higher among those with some college or an associate degree (*β = 19.27*, p < 0.01), 25.86 percentage points higher among those with a bachelor’s degree (*β = 25.86*, p < 0.001), and 28.99 percentage points higher among those with a master’s degree or higher (*β = 28.99*, p < 0.001). Other demographic characteristics, including age, gender, marital status, and employment status, are not significantly associated with cannabis legal sourcing.

To address potential heterogeneity, we re-estimate the model using restricted samples. First, we limit the sample to Ohio and its neighboring states—Pennsylvania, Indiana, West Virginia, Kentucky, Michigan, and Illinois—to account for regional factors and potential cross-border dynamics. Second, we restrict the sample to states with mixed cannabis policies prior to treatment. As shown in Table 3, subsample analyses yield positive but statistically insignificant estimates, likely reflecting reduced statistical power in smaller samples, rather than a reversal of the main pattern. Table A.2 shows the estimated coefficients for control variables.

## 4. DISCUSSIONS

This study provides new evidence on whether recreational cannabis legalization induces substitution from illicit to legal markets by examining individual-level sourcing behavior in a national panel setting. Using three waves of nationally representative survey data and a difference-in-differences framework, we find that legalization in Ohio increased the proportion of cannabis obtained from legal sources by approximately 5.3 percentage points in the short run. The finding is consistent with the policy objective of shifting demand toward regulated markets, but also suggests that legalization does not immediately eliminate reliance on illicit sources^9,23^.

This study contributes novel evidence to the cannabis legalization literature by examining an outcome that has received little direct empirical attention, namely the extent to which legalization shifts cannabis sourcing from illicit to legal sources. Existing studies have largely focused on how legalization affects cannabis prevalence, frequency of use, and initiation, typically using repeated cross-sectional or quasi-experimental designs and generally reporting modest increases in adult use alongside mixed or null effects on youth consumption^9,14–19,23^. While informative about consumption patterns, these studies do not directly assess whether legalization achieves one of its central policy objectives, reallocating demand away from illicit markets and into regulated legal channels. By focusing explicitly on legal sourcing rather than prevalence outcomes, and by directly measuring individuals’ reliance on legal versus illegal source, our study provides new and policy-relevant evidence on a key mechanism through which legalization is intended to generate public health, regulatory, and fiscal benefits.

More broadly, we find that legal sourcing differs substantially across policy environments, with individuals in fully legalized states reporting markedly higher reliance on legal markets. Our results suggest that legalization alone may be insufficient to fully displace illicit markets, particularly in the short run following retail market entry. While legalization creates a legal alternative, the persistence of illicit sourcing indicates that consumers’ decisions depend not only on legal status but also on the relative attractiveness of legal markets along dimensions such as price, accessibility, product variety, and perceived convenience^9,12,23,29,30^. This finding has important policy implications. While legalization establishes a legal alternative, it is unlikely to substantially undermine illicit supply on its own. Accelerating substitution toward regulated channels may require complementary policies that reduce effective price differentials, expand legal retail access, and ensure that legal products align with consumer preferences in terms of potency, variety, and convenience.

Our study also underscore that legalization reshapes cannabis markets primarily through changes in sourcing behavior rather than immediate changes in overall consumption. Prior research has documented shifts in expenditures, market composition, consumer attitudes toward legal versus illegal products, and spillover effects on co-use with substances such as cigarettes^20–22,24^. In contrast, our results provide direct evidence that legalization can meaningfully reallocate how consumers obtain cannabis, highlighting the importance of distinguishing between consumption responses and sourcing responses when evaluating legalization outcomes. From a policy perspective, this distinction is critical. Public health protections, regulations, and tax revenues depend on whether consumption occurs within the legal market, not simply on how much cannabis is consumed^7,9,29–31^.

The heterogeneity analyses indicate that the short-run effects of legalization are not uniform across policy environments and population subgroups. Although the estimated legalization effect remains positive when the sample is restricted to neighboring states or to states with mixed cannabis policies, these estimates remain positive but are not statistically significant, likely reflecting reduced statistical power in smaller subsamples. The results reveal substantial heterogeneity across demographic characteristics, with legal sourcing systematically lower among Black non-Hispanic respondents and substantially higher among individuals with greater educational attainment. These patterns suggest that consumers’ transitions into legal markets may depend on socioeconomic factors such as information, trust in regulated systems, and access to legal retail channels. These findings imply that legalization may unevenly shift sourcing behavior across demographic groups, potentially limiting the extent to which regulated markets displace illicit supply in the short run^12,14,21,24,32^.

Several limitations should be noted. First, the outcome measure is based on self-reported purchasing behavior, which may be subject to reporting error or social desirability bias. However, the use of a continuous percentage measure allows respondents to report partial reliance on legal sources, which likely reduces misclassification relative to binary measures of legal versus illegal use. Second, the analysis captures short-run responses following legalization; longer-run substitution patterns may differ as legal markets mature, prices adjust, and retail availability expands. Third, the number of Ohio respondents is relatively small, which may limit precision in subgroup analyses.

Despite these limitations, the study provides timely and policy-relevant evidence on the extent to which legalization shifts cannabis purchases toward regulated markets. The results suggest that legalization can shift consumer behavior toward legal markets, but that the transition is incomplete in the early post-legalization period. Additionally, the observed shifts in sourcing are uneven across demographic groups, with substantially lower legal-market reliance among Black non-Hispanic respondents and markedly higher reliance among individuals with greater educational attainment, highlighting that legalization does not affect all consumers equally. By directly measuring the share of cannabis obtained from legal markets, this study contributes new evidence on substitution between illicit and regulated channels. Future research should examine longer-run effects, explore mechanisms underlying incomplete substitution, and assess how sourcing responses vary across different policy environments and market conditions.

## Figures and Tables

**Table 1.** Summary Statistics for Study Variables

Variable	Mean (SD)/Percentage
***Dependent Variables***	
Proportion of Legal Cannabis Purchase	77.63 (37.64)
***Independent Variables***	
Period	
Pre-treatment	33.33%
Post-treatment	66.67%
Treated (in Ohio)	
Yes	4.15%
No	95.85%
Legalization Status	
Fully Illegal	6.17%
Fully legal	67.94%
Mixed	25.89%
Pre-Treatment Legalization Status	
Fully Illegal	6.17%
Fully legal	63.78%
Mixed	30.05%
***Demographic Variables***	
Age	50.23 (15.29)
Gender	
Male	60.55%
Female	39.45%
Race/Ethnicity	
White, Non-Hispanic	76.82 %
Black, Non-Hispanic	8.48 %
Other, Non-Hispanic	1.04%
Hispanic	10.03%
2+ Races, Non-Hispanic	3.63%
Marital Status	
Not married	50.75%
Married	49.25%
Education	
No high school diploma or GED	4.27%
High school graduate (or equivalent GED)	16.90%
Some college or Associate’s degree	34.89%
Bachelor’s degree	28.26%
Master’s degree or higher	15.69%
Employment	
Working full-time	53.69%
Working part-time	13.84%
Not working	32.47%
**Observations**	**1,734**

**Table 2.** Difference-in-Differences Estimates of Ohio Recreational Cannabis Legalization on Legal Sourcing

	Proportion of Legal	Cannabis Purchase
	(1)	(2)
Treatment State (base: Non-Ohio)		
Ohio	8.423*	8.887*
	(3.941)	(4.389)
Post × Treatment	5.330***	5.305***
	(1.223)	(1.282)
Pre-Treatment Legalization Status (base: Fully illegal)		
Fully legal	33.626***	31.648***
	(3.463)	(3.995)
Mixed	11.776*	11.289
	(4.884)	(5.656)
Control Variables	Not Included	Included
Observations	1,734	1,734

***Notes***: All models include wave fixed effects; therefore, the main effect of the post-legalization period is absorbed and not separately estimated. Standard errors in parentheses.

* *p* < 0.05,

** *p* < 0.01,

*** *p* < 0.001

**Table 3.** Difference-in-Differences Estimates in Neighboring-State and Mixed-Policy Subsamples

	Proportion of Legal	Cannabis Purchase
	(1)	(2)
Treatment State (base: Non-Ohio)		
Ohio	−3.128	9.211*
	(3.337)	(4.367)
Post × Treatment	2.186	3.377
	(2.929)	(2.383)
Pre-Treatment Legalization Status (base: Fully legal)		
Mixed	−10.194**	-
	(2.500)	-
Control Variables	Not Included	Included
Observations	385	521

***Notes***: Column (1) restricts the sample to Ohio and neighboring states (Pennsylvania, Indiana, West Virginia, Kentucky, Michigan, and Illinois). Column (2) restricts the sample to states with mixed cannabis policies prior to treatment (medical cannabis legal, recreational cannabis illegal). All models include wave fixed effects; therefore, the main effect of the post-legalization period is absorbed and not separately estimated. Standard errors in parentheses.

* *p* < 0.05,

** *p* < 0.01,

*** *p* < 0.001

## Data Availability

The data analyzed in this study are not publicly available due to data use restrictions. Access may be granted to qualified researchers upon reasonable request.

## Appendix

**Table A.1:** Difference-in-Differences Estimates of Ohio Recreational Cannabis Legalization on Legal Sourcing

	Proportion of Legal Cannabis Purchase
Age	−0.112
	(0.091)
Gender (base: Male)	
Female	3.400
	(2.612)
Race/Ethnicity (base: White, Non-Hispanic)	
Black, Non-Hispanic	−16.939**
	(4.988)
Other, Non-Hispanic	8.820***
	(2.058)
Hispanic	0.826
	(3.873)
2+ Races, Non-Hispanic	−1.981
	(5.378)
Marital Status (base: Not married)	
Married	2.936
	(2.830)
Education (base: No high school diploma or GED)	
High school graduate (or the equivalent GED)	15.677*
	(5.920)
Some college or Associate’s degree	19.273**
	(6.356)
Bachelor’s degree	25.861***
	(5.742)
Master’s degree or higher	28.991***
	(6.367)
Employment (base: Working full-time)	
Working part-time	0.378
	(4.216)
Not working	3.210
	(3.223)
Observations	1734

Standard errors in parentheses

* *p* < 0.05,

** *p* < 0.01,

*** *p* < 0.001

**Table A.2:** Difference-in-Differences Estimates in Neighboring-State and Mixed-Policy Subsamples

	Proportion of Legal	Cannabis Purchase
	(1)	(2)
Age	−0.031	0.093
	(0.186)	(0.198)
Gender (base: Male)		
Female	−2.551	0.504
	(4.991)	(5.129)
Race/Ethnicity (base: White, Non-Hispanic)		
Black, Non-Hispanic	−12.219*	−29.977**
	(4.937)	(9.344)
Hispanic	4.792	−4.916
	(6.029)	(9.156)
2+ Races, Non-Hispanic	16.145*	−9.490
	(4.513)	(13.271)
Marital Status (base: Not married)		
Married	1.349	−4.400
	(3.816)	(4.816)
Education (base: No high school diploma or GED)		
High school graduate (or the equivalent GED)	18.891	15.806
	(26.939)	(13.264)
Some college or Associate’s degree	22.865	19.773
	(25.151)	(12.118)
Bachelor’s degree	28.761	32.799*
	(28.097)	(12.117)
Master’s degree or higher	33.627	41.164***
	(26.684)	(9.499)
Employment (base: Working full-time)		
Working part-time	5.436	4.957
	(6.932)	(7.351)
Not working	1.584	0.921
	(5.497)	(6.883)
Observations	385	521

## References

[1] Lampe Joanna R., Sacco Lisa N., Sheikh Hassan Z.. The Federal Status of Marijuana and the Policy Gap with States. legislation. 2024. Available from: https://www.congress.gov/crs-product/IF12270 [Last accessed: 1/5/2026].

[2] Pacula RL, Sevigny EL. Marijuana Liberalizations Policies: Why We Can’t Learn Much from Policy Still in Motion. J Policy Anal Manage 2014;33(1):212–221; doi: 10.1002/pam.21726. 24358530 PMC4051884

[3] National Conference of State Legislatures. State Medical Cannabis Laws. 2025. Available from: https://www.ncsl.org/health/state-medical-cannabis-laws [Last accessed: 1/6/2026].

[4] Pacula RL, Smart R. Medical Marijuana and Marijuana Legalization. Annu Rev Clin Psychol 2017;13:397–419; doi: 10.1146/annurev-clinpsy-032816-045128. 28482686 PMC6358421

[5] Lampe Joanna R.. State Marijuana “Legalization” and Federal Drug Law: A Brief Overview for Congress. legislation. 2024. Available from: https://www.congress.gov/crs-product/LSB10482 [Last accessed: 1/6/2026].

[6] LA Department of Cannabis Regulation. 2024 US Cannabis Sales | Cannabis Regulation. 2025. Available from: https://cannabis.lacity.gov/articles/2024-us-cannabis-sales [Last accessed: 1/6/2026].

[7] National Academies of Sciences, Engineering, and Medicine. Cannabis Policy Impacts Public Health and Health Equity. National Academies Press: Washington, D.C.; 2024.; doi: 10.17226/27766. 39602559

[8] American Public Health Association. Regulating Commercially Legalized Marijuana as a Public Health Priority. 2014. Available from: https://www.apha.org/policy-and-advocacy/public-health-policy-briefs/policy-database/2015/01/23/10/17/regulating-commercially-legalized-marijuana-as-a-public-health-priority?utm_source=chatgpt.com [Last accessed: 1/9/2026].

[9] Smart R, Pacula RL. Early evidence of the impact of cannabis legalization on cannabis use, cannabis use disorder, and the use of other substances: Findings from state policy evaluations. Am J Drug Alcohol Abuse 2019;45(6):644–663; doi: 10.1080/00952990.2019.1669626. 31603710 PMC6934162

[10] Sutcliffe OB, Erridge S, Martinez J. The Hidden Dangers of Illicit Cannabis. Report. Curaleaf International; 2024.

[11] Botelho D, Boudreau A, Rackov A, Analysis of Illicit and Legal Cannabis Products for a Suite of Chemical and Microbial Contaminants. 2021.

[12] Robertson K, Thyne M. Legalization of recreational cannabis: Facilitators and barriers to switching from an illegal to a legal source. Prev Med Rep 2021;24:101639; doi: 10.1016/j.pmedr.2021.101639. 34976690 PMC8683989

[13] Donnan J, Shogan O, Bishop L, Characteristics that influence purchase choice for cannabis products: a systematic review. J Cannabis Res 2022;4:9; doi: 10.1186/s42238-022-00117-0. 35105374 PMC8805380

[14] Hyatt AS, Flores MW, Johnson J, Cannabis Use Among Individuals With Psychosis After State-Level Commercial Cannabis Legalization. JAMA Psychiatry 2025; doi: 10.1001/jamapsychiatry.2025.2539. PMC12509079 41060643

[15] Hyatt AS, Overhage L, Cook BL. Use of Tobacco and Cannabis Following State-Level Cannabis Legalization. JAMA Netw Open 2025;8(7):e2520093; doi: 10.1001/jamanetworkopen.2025.20093. 40643912 PMC12254894

[16] Manthey J, Hayer T, Jacobsen B, Effects of legalizing cannabis. n.d.

[17] Mennis J, McKeon TP, Stahler GJ. Recreational cannabis legalization alters associations among cannabis use, perception of risk, and cannabis use disorder treatment for adolescents and young adults. Addictive Behaviors 2023;138:107552; doi: 10.1016/j.addbeh.2022.107552. 36413909

[18] Montgomery BW, Roberts MH, Margerison CE, Estimating the effects of legalizing recreational cannabis on newly incident cannabis use. PLOS ONE 2022;17(7):e0271720; doi: 10.1371/journal.pone.0271720. 35862417 PMC9302774

[19] Rotermann M. What has changed since cannabis was legalized? Health Reports 2020;31(2):11–20. 32073644 10.25318/82-003-x202000200002-eng

[20] McDonald AJ, Cooper A, Doggett A, Association of recreational cannabis legalization with changes in medical, illegal, and total cannabis expenditures in Canada. International Journal of Drug Policy 2025;139:104793; doi: 10.1016/j.drugpo.2025.104793. 40188703

[21] Donnan J, Shogan O, Bishop L, Drivers of purchase decisions for cannabis products among consumers in a legalized market: a qualitative study. BMC Public Health 2022;22(1):368; doi: 10.1186/s12889-021-12399-9. 35189856 PMC8860259

[22] Xing J, Shi Y. Cannabis consumers’ preferences for legal and illegal cannabis: evidence from a discrete choice experiment. BMC Public Health 2024;24(1):2397; doi: 10.1186/s12889-024-19640-1. 39227852 PMC11373389

[23] Meinhofer A, Rubli A. Illegal drug market responses to state recreational cannabis laws. Addiction 2021;116(12):3433–3443; doi: 10.1111/add.15517. 33998087 PMC8578142

[24] Weinberger AH, Wyka K, Kim JH, A difference-in-difference approach to examining the impact of cannabis legalization on disparities in the use of cigarettes and cannabis in the United States, 2004–17. Addiction 2022;117(6):1768–1777; doi: 10.1111/add.15795. 34985165 PMC12445298

[25] Xu L, He Y, Park H, How tax structures for retail cannabis shape cannabis use among youth and young adults: evidence from a volumetric choice experiment. Eur J Health Econ 2025; doi: 10.1007/s10198-025-01875-3. PMC12922899 41460273

[26] Ohio Secretary of State. 2023 Official Election Results. 2023. Available from: https://www.ohiosos.gov/elections/election-results-and-data/2023-official-election-results/[Last accessed: 1/6/2026].

[27] The Ohio House of Representatives. Adult Use Sales Commence in Ohio. 2024. Available from: https://www.ohiohouse.gov/members/jamie-callender/news/adult-use-sales-commence-in-ohio-121466 [Last accessed: 1/6/2026].

[28] Benson C. Adult-Use Marijuana Sales to Begin in Ohio Tomorrow. 2024. Available from: https://www.upi.com/Top_News/US/2024/08/05/Ohio-Commerce-Department-buy-legal-marijuana-cannabis/8711722885337/[Last accessed: 1/6/2026].

[29] Caulkins JP, Kilmer B. Considering Marijuana Legalization Carefully: Insights for Other Jurisdictions from Analysis for Vermont. Addiction 2016. 10.1111/add.13289 28075542

[30] Hansen B, Miller K, Weber C. FEDERALISM, PARTIAL PROHIBITION, AND CROSS-BORDER SALES: EVIDENCE FROM RECREATIONAL MARIJUANA. n.d.

[31] Pacula RL, Kilmer B, Wagenaar AC, Developing Public Health Regulations for Marijuana: Lessons From Alcohol and Tobacco. Am J Public Health 2014;104(6):1021–1028; doi: 10.2105/AJPH.2013.301766. 24825201 PMC4062005

[32] Fataar F, Goodman S, Wadsworth E, Consumer perceptions of ‘legal’ and ‘illegal’ cannabis in US states with legal cannabis sales. Addictive Behaviors 2021;112:106563; doi: 10.1016/j.addbeh.2020.106563. 32768793

